# Evaluation of the Effect of Topical 0.2% Hyaluronic Acid Gel on Postoperative Pain and Wound Healing Following Gingival Depigmentation: A Prospective Clinical Study

**DOI:** 10.7759/cureus.111218

**Published:** 2026-06-20

**Authors:** Parween Jamil, Anupama Lakharwal, Smruti R Thoppil, Murali Krishna Tupili, Sanpreet S Sachdev, Mariyam S Momin

**Affiliations:** 1 Department of Periodontology and Oral Implantology, Government Dental College and Hospital Shereen Bagh, Srinagar, IND; 2 Department of Periodontology, Sri Sai College of Dental Surgery, Vikarabad, IND; 3 Department of Oral Pathology and Microbiology, Bharati Vidyapeeth (Deemed to be University) Dental College and Hospital, Mumbai, IND; 4 Department of Periodontology, M. A. Rangoonwala College of Dental Sciences and Research Centre, Pune, IND

**Keywords:** gingival depigmentation, gingival hyperpigmentation, hyaluronic acid, postoperative pain, wound healing

## Abstract

Introduction

Gingival hyperpigmentation is a common esthetic concern that may negatively affect the appearance of the smile and patient confidence. Gingival depigmentation procedures often result in postoperative discomfort and delayed healing due to the exposed connective tissue wound surface. This study aimed to evaluate the effect of topical application of 0.2% hyaluronic acid gel on postoperative pain and wound healing following gingival depigmentation surgery.

Materials and methods

This prospective clinical study included 25 systemically healthy individuals with physiologic gingival hyperpigmentation who underwent gingival depigmentation using the conventional scalpel technique. Following the surgical procedure, topical 0.2% hyaluronic acid gel was applied to the surgical site along with periodontal dressing placement. Postoperative pain was assessed using the visual analog scale (VAS) on days 1, 3, 7, and 14, whereas wound healing was evaluated using the healing index on days 3, 7, 14, and 21. Statistical analysis was performed using the Friedman test followed by post hoc analysis with the Wilcoxon signed-rank test for ordinal data. A p-value less than 0.05 was considered statistically significant.

Results

The median postoperative pain score progressively decreased from 4 (interquartile range (IQR): 3-5) on day 1 to 2 (IQR: 1-3) on day 3, 1 (IQR: 0-2) on day 7, and 0 (IQR: 0-0) on day 14. Friedman test analysis demonstrated a statistically significant difference in wound healing scores across the postoperative follow-up period (χ² = 67.54, p < 0.001). Wound healing scores showed continuous improvement during follow-up, increasing from a median score of 3 (IQR: 3-4) on day 3 to 7 (IQR: 6-7) on day 21. The improvement in the healing scores was statistically significant (χ² = 67.54, p < 0.001). Post hoc analysis further revealed that pain scores showed a significant reduction across follow-up periods, with the greatest decrease observed between day 1 and day 14 (p < 0.001). Wound healing scores improved significantly over time, particularly from day 1 to later follow-up visits and between day 3 and day 14/21 (all p ≤ 0.001), while changes between the final assessment intervals were not statistically significant after Bonferroni correction.

Conclusion

Topical application of 0.2% hyaluronic acid gel following gingival depigmentation was associated with favorable postoperative healing outcomes and an overall reduction in pain scores during the healing period. Hyaluronic acid appears to be a safe and clinically useful adjunct in the postoperative management of gingival depigmentation procedures. Further controlled studies are warranted to confirm its effectiveness in improving patient comfort and wound healing.

## Introduction

Gingival hyperpigmentation is a common esthetic concern caused primarily by excessive melanin deposition in the basal and suprabasal layers of the gingival epithelium [1]. Although physiologic gingival pigmentation is not considered a pathological condition, many individuals seek treatment because of compromised smile esthetics and psychological discomfort. Therefore, gingival depigmentation procedures are frequently performed in periodontal practice to improve gingival appearance and patient confidence [1,2].

Various surgical techniques have been advocated for gingival depigmentation, including scalpel surgery, electrosurgery, cryosurgery, laser therapy, and abrasion [2,3]. Among these, the conventional scalpel technique remains widely used because of its simplicity, cost-effectiveness, and satisfactory clinical outcome [4]. However, this procedure leaves a denuded connective tissue surface that may result in postoperative pain, delayed healing, discomfort during mastication, and increased susceptibility to local trauma during the healing phase [5]. Therefore, adjunctive therapeutic agents that can accelerate tissue repair and reduce postoperative morbidity are of considerable clinical interest.

Hyaluronic acid (HA) is a naturally occurring, high-molecular-weight polysaccharide found in connective tissues, synovial fluid, and extracellular matrices. It plays a vital role in tissue hydration, cell migration, angiogenesis, and inflammatory response modulation during wound healing. Owing to its anti-inflammatory, bacteriostatic, antioxidant, and proangiogenic properties, HA has been increasingly utilized in periodontal and oral surgical procedures to promote soft-tissue healing and improve patient comfort [6]. The topical application of 0.2% HA gel has shown promising results in enhancing epithelialization, reducing inflammation, and minimizing postoperative pain in various oral wound conditions [7].

Despite the growing use of hyaluronic acid in periodontal therapy, there is limited evidence regarding its effectiveness in gingival depigmentation procedures. Therefore, evaluating its role in improving postoperative healing outcomes may provide valuable clinical guidance for enhancing patient care and comfort after periodontal surgery. The present study aimed to evaluate postoperative pain and wound healing following the topical application of 0.2% hyaluronic acid (HA) gel after gingival depigmentation surgery. The objectives were to assess postoperative pain levels, evaluate wound healing outcomes, and analyze the healing pattern at different postoperative follow-up intervals.

## Materials and methods

Study design and setting

This prospective clinical study was conducted at the Department of Periodontology and Oral Implantology, Government Dental College and Hospital, Srinagar, India, from September 2024 to March 2025. The study protocol was explained to all participants, and written informed consent was obtained prior to their enrollment. Ethical clearance for the study was obtained from the Institutional Ethics Committee (GDC/Perio/Eth Committee/1799) before the commencement of the study.

Sample size estimation

The sample size was calculated using G*Power software version 3.1.9.7 (Department of Experimental Psychology, Heinrich Heine University, Düsseldorf, Germany. As no previous single-arm studies reporting the required parameters were available, the effect size was derived from the difference in postoperative outcomes reported by Fareed et al. [6]. Based on a large effect size (d = 0.80), a significance level of 5% and study power of 80%, the minimum required sample size was calculated to be 21 participants. To compensate for possible dropouts, 25 participants were recruited.

Study population

A total of 25 systemically healthy individuals with physiological gingival hyperpigmentation were included in the study. Participants aged between 20 and 40 years with esthetic concerns related to gingival pigmentation on the facial aspect of the anterior gingiva were recruited from the outpatient section of the department.

Inclusion and exclusion criteria

Systemically healthy individuals with plaque and gingival index scores ≤1, adequate attached gingival width, and clinically evident physiological gingival hyperpigmentation were included in the study [8]. Patients with a history of periodontal surgery within the previous six months, smokers, pregnant or lactating women, individuals receiving antibiotics or anti-inflammatory medications, and those with systemic diseases or conditions affecting wound healing were excluded from the study.

Clinical armamentarium and materials used

The clinical armamentarium used in the study included a mouth mirror, explorer, UNC-15 periodontal probe, tweezers, ultrasonic scaler with tips, Gracey curettes, cotton swabs, gauze pieces, disposable syringes, BP blade handle, and BP blade. 15. The HA material used in the study was 0.2% HA gel (Gengigel, Ricerfarma S.r.l., Milan, Italy). A periodontal dressing was placed following the procedure to protect the surgical site during the initial healing period.

Preoperative procedure

All participants underwent Phase I periodontal therapy one week prior to the surgical procedure, which included oral prophylaxis and oral hygiene instruction. Baseline clinical examination was performed, and demographic details were recorded. Gingival pigmentation scores were assessed before the procedure.

Surgical procedure

**Figure 1:**
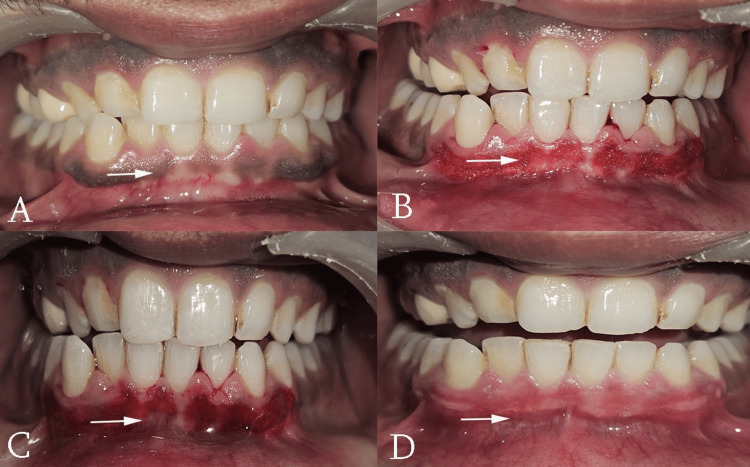
Clinical photographs illustrating the surgical depigmentation procedure and postoperative healing (A) Preoperative image showing physiologic gingival melanin pigmentation in the mandibular anterior region. (B) Conventional scalpel surgical depigmentation procedure being performed. (C) Application of 0.2% hyaluronic acid gel over the surgically treated gingival surface immediately after depigmentation. (D) Postoperative healing of the surgical site demonstrating satisfactory epithelialization and tissue recovery during follow-up. The white arrow indicates the treated gingival area in each image.

All surgical procedures were performed under aseptic conditions by a single operator. Following the administration of local anesthesia, gingival depigmentation was performed using the conventional scalpel technique with a BP blade no. 15. The pigmented epithelium, along with a thin layer of underlying connective tissue, was carefully removed from the facial aspect of the gingiva until the exposed connective tissue appeared free of pigmentation. After achieving adequate hemostasis, topical 0.2% HA gel was evenly applied over the depigmented surgical site. A periodontal dressing was placed to protect the wound. The participants were instructed to avoid trauma to the surgical site and were provided with standard postoperative instructions and medications (Figure 1).

Clinical evaluation

Postoperative pain and wound healing were evaluated at different follow-up periods. Pain assessment was performed using the visual analog scale (VAS) on postoperative days 1, 3, 7, and 14 [9]. Wound healing was assessed using the Early Wound Healing Score (EHS) proposed by Marini et al. [10] on postoperative days 3, 7, 14, and 21. The EHS is a validated clinical index used to evaluate early wound healing based on clinical parameters related to tissue healing and inflammatory response. The total EHS ranges from 0 to 10, with higher scores indicating better wound healing. All clinical assessments were performed by the same examiner to minimize inter-examiner variability.

Statistical analysis

Statistical analyses were performed using IBM SPSS Statistics software version 23.0 (IBM Corp., Armonk, NY, USA). Data were assessed for normality using the Shapiro-Wilk test. Continuous variables are expressed as medians and interquartile ranges (IQR), and categorical variables are presented as frequencies and percentages. Changes in postoperative pain and wound healing scores over different follow-up periods were analyzed using the Friedman test followed by post hoc analysis with the Wilcoxon signed-rank test. Statistical significance was set at p < 0.05.

## Results

A total of 25 participants completed the study and were included in the final analyses. The demographic and baseline clinical characteristics of the participants are shown in Table 1. The median age of the participants was 28.0 years (IQR: 24.0-33.0 years). Among the study participants, 13 (52.0%) were men and 12 (48.0%) were women. The median baseline gingival pigmentation score was 3.0 (IQR: 2.0-3.0). The distribution of surgical sites showed that 14 (56.0%) procedures were performed in the maxillary arch and 11 (44.0%) in the mandibular arch. These findings indicate a relatively homogeneous study population at baseline.

**Table 1:** Demographic and baseline characteristics of participants (n = 25) Data are presented as median (interquartile range) for continuous variables and frequency (percentage) for categorical variables. IQR: interquartile range

Variable	Measure	0.2% hyaluronic acid gel group (n= 25)
Age (years)	Median (IQR)	28.0 (24.0-33.0)
Sex (Male/Female)	n (%)	13 (52.0%) / 12 (48.0%)
Gingival pigmentation score	Median (IQR)	3.0 (2.0-3.0)
Surgical site (Maxillary/Mandibular)	n (%)	14 (56.0%) / 11 (44.0%)

Postoperative pain scores, assessed using the VAS at different follow-up intervals, are presented in Table 2. The median postoperative pain score on day 1 was 4 (IQR: 3-5), which gradually reduced to 2 (IQR: 1-3) on day 3, 1 (IQR: 0-2) on day 7, and 0 (IQR: 0-0) on day 14. The progressive reduction in pain scores over the postoperative period indicated gradual resolution of discomfort following the topical application of 0.2% HA gel after gingival depigmentation.

**Table 2:** Postoperative pain scores at different follow-up intervals following the application of 0.2% hyaluronic acid gel Data are presented as median (interquartile range). Postoperative pain scores were assessed using the visual analog scale (VAS). IQR: interquartile range

Postoperative day	0.2% hyaluronic acid gel group, Median (IQR)
Day 1	4 (3-5)
Day 3	2 (1-3)
Day 7	1 (0-2)
Day 14	0 (0-0)

The within-group analysis of postoperative pain scores over time using the Friedman test is shown in Table 3. A statistically significant reduction in pain scores was observed across all postoperative follow-up periods (χ² = 64.82, p < 0.001). The findings demonstrated that pain levels progressively decreased during the healing period following 02% HA gel application.

**Table 3:** Within-group comparison of postoperative pain scores over time Data are presented as median (interquartile range). Within-group comparisons across follow-up periods were analyzed using the Friedman test. *p-value < 0.05 was considered statistically significant. IQR: interquartile range, df: degree of freedom

Pain score	Day 1	Day 3	Day 7	Day 14	Test value (df)	p-value
0.2% hyaluronic acid gel, Median (IQR)	4 (3-5)	2 (1-3)	1 (0-2)	0 (0-0)	64.82 (3)	< 0.001*

Wound healing outcomes assessed using EHS at different postoperative intervals are shown in Table 4. The median wound healing score on day 3 was 3 (IQR: 3-4), which improved to 4 (IQR: 4-5) on day 7. Further improvement was observed on day 14, with a median score of 6 (IQR: 4-6), and complete or near-complete healing was achieved by day 21, with a median score of 7 (IQR: 6-7). These findings suggest a progressive improvement in soft tissue healing following the topical application of 0.2% HA gel.

**Table 4:** Wound healing scores at different postoperative follow-up intervals following application of 0.2% hyaluronic acid gel Data are presented as median (interquartile range). Wound healing was assessed using the early wound healing score (EHS) by Marini et al. [10]. IQR: interquartile range

Postoperative day	0.2% hyaluronic acid gel, Median (IQR)
Day 3	3 (3-4)
Day 7	4 (4-5)
Day 14	6 (4-6)
Day 21	7 (6-7)

The within-group comparison of wound healing scores over time using the Friedman test is presented in Table 5. A statistically significant improvement in the wound healing scores was observed throughout the postoperative follow-up period (χ² = 67.54, p < 0.001). The results indicated favorable healing progression and enhanced tissue repair following gingival depigmentation treatment with topical 0.2% HA gel.

**Table 5:** Within-group comparison of wound healing scores over time Data are presented as median (interquartile range). Within-group comparisons across follow-up intervals were analyzed using the Friedman test. *p-value < 0.05 was considered statistically significant. IQR: interquartile range, df: degree of freedom

Wound healing score	Day 3	Day 7	Day 14	Day 21	Test value (df)	p-value
0.2% hyaluronic acid gel, Median (IQR)	3 (3-4)	4 (4-5)	6 (4-6)	7 (6-7)	67.54 (3)	< 0.001*

Post hoc pairwise comparisons using the Wilcoxon signed-rank test with Bonferroni correction demonstrated a significant reduction in pain scores over time (Table 6). Pain scores decreased significantly between day 1 and day 3, day 7, and day 14 (all p < 0.001), with the greatest reduction observed between day 1 and day 14 (mean difference = 4.0). Significant reductions were also noted between day 3 and day 7 (p = 0.008) and day 3 and day 14 (p < 0.001), while the difference between day 7 and day 14 was not significant after Bonferroni correction (p = 0.009). Similarly, wound healing scores improved significantly from day 1 to subsequent follow-up periods and between day 3 and day 14/21 (all p ≤ 0.001). However, the improvement between day 14 and day 21 was not statistically significant after correction (p = 0.021). 

**Table 6:** Post hoc pairwise comparisons using the Wilcoxon signed-rank test with Bonferroni correction for pain and wound healing scores *p < 0.008 is considered significant after Bonferroni correction.

Comparison	Pain score			Wound healing score		
	Mean difference	Z value	p-value	Mean difference	Z value	p-value
Day 1 vs Day 3	1.8	-3.85	< 0.001*	0.8	-3.85	< 0.001*
Day 1 vs Day 7	2.8	-4.20	< 0.001*	1.8	-5.10	< 0.001*
Day 1 vs Day 14	4.0	-4.45	< 0.001*	2.8	-5.95	< 0.001*
Day 3 vs Day 7	0.9	-3.10	0.008	0.8	-3.25	0.001*
Day 3 vs Day 14	1.9	-3.95	< 0.001*	1.8	-4.85	< 0.001*
Day 7 vs Day 14	0.8	-2.65	0.009	0.8	-2.30	0.021

## Discussion

Gingival hyperpigmentation is a common esthetic concern that frequently affects patient confidence and smile esthetics. Although gingival depigmentation procedures are effective in improving gingival appearance, they often result in postoperative discomfort and delayed healing due to the creation of a raw connective tissue wound surface. Therefore, adjunctive agents that can accelerate healing and minimize postoperative pain are of considerable clinical importance. This prospective clinical study evaluated the effect of topical application of 0.2% HA gel on postoperative pain and wound healing following gingival depigmentation surgery.

The findings of the present study demonstrated a progressive reduction in postoperative pain scores during the healing period of the study. Pain intensity was highest on the first postoperative day and gradually decreased over subsequent follow-up visits, reaching minimal or no pain by day 14. The statistically significant reduction in pain scores observed over time may be attributed to HA’s anti-inflammatory and hygroscopic properties. HA forms a protective viscoelastic layer over the wound surface, reducing tissue dehydration and shielding exposed nerve endings from external irritation [7,11]. In addition, HA modulates inflammatory mediators and reduces local tissue edema, which may contribute to decreased postoperative discomfort [11].

The favorable reduction in postoperative pain observed in the present study is in accordance with the findings reported by Kale et al., who demonstrated significantly lower postoperative pain scores in patients receiving topical 0.2% HA gel following gingival depigmentation [12]. Similarly, Fareed et al. reported reduced postoperative discomfort and improved patient acceptance with HA use after depigmentation surgery [6]. Previous studies evaluating HA in oral surgery and periodontal procedures have demonstrated its effectiveness in reducing inflammation, pain perception, and tissue irritation during healing [13].

The present study also demonstrated a progressive improvement in the wound healing scores throughout the postoperative period. Healing scores increased consistently from day 3 to day 21, indicating favorable tissue repair and epithelialization. The enhanced healing response observed with topical HA application may be explained by its biological role in extracellular matrix organization, angiogenesis, fibroblast migration, and collagen deposition. HA acts as a temporary scaffold during the initial stages of wound healing and promotes cellular proliferation and the formation of granulation tissue. Its hydrophilic nature also maintains tissue hydration, facilitating epithelial migration and faster wound closure [13,14].

The improved wound healing outcomes observed in the present study are consistent with those of earlier investigations. Kale et al. demonstrated superior epithelialization and improved healing indices with topical HA application following gingival depigmentation [12]. Similar beneficial effects of HA on soft tissue healing have been reported in studies involving periodontal surgery, extraction sockets, and oral ulcerative lesions. Gontiya and Galgali reported accelerated tissue repair and improved clinical healing with topical hyaluronic acid in recurrent aphthous ulcers [15], whereas Pilloni et al. observed enhanced periodontal wound healing associated with HA application [16]. These findings collectively support the regenerative and anti-inflammatory potential of HA in the management of oral soft tissues.

Another possible explanation for the favorable healing response observed in the present study may be related to the bacteriostatic and antioxidant properties of HA. HA inhibits bacterial colonization and neutralizes free radicals generated during inflammatory reactions, thereby minimizing tissue destruction and supporting rapid healing [11]. Furthermore, HA promotes neovascularization and improves oxygen and nutrient diffusion to the wound site, which may contribute to earlier tissue maturation and improved clinical healing scores [13].

The findings of the present study have important clinical implications. Topical application of 0.2% HA gel following gingival depigmentation appears to be a simple, non-invasive, and cost-effective adjunctive approach for improving postoperative patient comfort and enhancing wound healing. The material is highly biocompatible, easy to apply, and well-tolerated by patients, making it clinically useful in routine periodontal practices. Improved healing and reduced postoperative discomfort may also contribute to greater patient satisfaction and acceptance of esthetic periodontal treatment.

Despite these favorable outcomes, the present study has certain limitations. The study was conducted with a relatively small sample size at a single institution, which may limit the generalizability of the findings. In addition, the absence of a comparative control group in the present analysis limits the direct assessment of the magnitude of the benefit achieved with HA application. The follow-up duration was relatively short and primarily focused on early wound-healing outcomes. Future studies with larger multicentric samples, longer follow-up periods, and histological evaluation of tissue healing are recommended to further validate the clinical effectiveness of HA following gingival depigmentation procedures.

## Conclusions

In conclusion, the present study demonstrated that the topical application of 0.2% hyaluronic acid gel following gingival depigmentation resulted in a progressive reduction in postoperative pain and favorable wound healing outcomes during the healing period. The anti-inflammatory, hygroscopic, and pro-healing properties of hyaluronic acid may have contributed to enhanced tissue repair and improved patient comfort. Within the limitations of this study, topical hyaluronic acid gel appears to be a safe, effective, and clinically beneficial adjunct in the management of postoperative healing following gingival depigmentation procedures. Further long-term, multicenter studies with larger sample sizes are recommended to validate these findings and establish standardized clinical protocols for routine use in periodontal practice.
